# Anesthetic Challenges of Labor and Delivery in a Patient With Catecholaminergic Polymorphic Ventricular Tachycardia and Left Ventricular Non‐Compaction Cardiomyopathy

**DOI:** 10.1002/ccr3.70454

**Published:** 2025-04-21

**Authors:** Chase Jackson, Jibran Ikram, Jose L. Diz Ferre, Sabry Ayad

**Affiliations:** ^1^ Outcomes Research Consortium, Anesthesiology Department Cleveland Clinic Cleveland Ohio USA

**Keywords:** anesthesia management, catecholaminergic polymorphic ventricular tachycardia (CPVT), high‐risk pregnancy, left ventricular non‐compaction cardiomyopathy (LVNC), obstetric patients, peripartum cardiac risk

## Abstract

Catecholaminergic Polymorphic Ventricular Tachycardia (CPVT) and Left Ventricular Non‐Compaction Cardiomyopathy (LVNC) are inherited disorders that pose significant challenges in the obstetric population due to the potential exacerbation of ventricular arrhythmias and potentially lethal cardiac compromise during pregnancy and labor. This case report aims to provide insights into the anesthetic management of CPVT and LVNC in obstetric patients. Goals of management should include careful optimization of anesthesia and analgesia, particularly during the birthing process. The choice of anesthetic agents, particularly the avoidance of sympathomimetic drugs, is crucial in preventing catecholamine‐induced arrhythmias. Additionally, the utilization of regional anesthesia techniques, such as epidural analgesia, may offer benefits in mitigating sympathetic activation during labor while providing adequate pain relief. Close collaboration between obstetricians, anesthesiologists, and cardiologists is imperative to develop a comprehensive management plan tailored to the individual patient's needs.

**Summary**

- Catecholaminergic Polymorphic Ventricular Tachycardia (CPVT) and Left Ventricular Non‐Compaction Cardiomyopathy (LVNC) pose unique challenges in obstetric patients due to the risk of life‐threatening arrhythmias.
- Careful anesthetic management, including early epidural placement, avoidance of catecholaminergic agents, continuation of beta‐blockade, and multidisciplinary planning, is critical to ensure safe maternal and fetal outcomes during labor and delivery.

## 1 Introduction

CPVT is a rare condition characterized by adrenergic‐induced ventricular tachycardia that is usually bidirectional or polymorphic, is reproducible during exercise or heightened emotional states, etc., and can precipitate syncope and sudden cardiac death [1]. The prevalence of this condition is estimated to be 1/10,000 with both sexes equally affected [2]. The mortality rate of CPVT is high, reaching 30%–50% by age 35 if left untreated [3].

Additionally, LVNC is a congenital condition characterized by numerous endomyocardial trabeculations that form in the left ventricle. Although the incidence of this disorder is difficult to estimate due to nonspecific diagnostic criteria and is felt to be overdiagnosed, the incidence in children in some reports varied between 4.8% and 9.2%, making LVNC the third most common cardiomyopathy. The prevalence in adults is lower, approximately 4.1%–5% [4].

Our aim is to highlight the anesthetic management of labor and delivery of a patient with CPVT. In our case, this context is worsened by preexisting LVNC, which can result in ventricular arrhythmias or even sudden cardiac death after potential endogenous catecholamine release from stress, pain, or anxiety‐type states. Anesthetic management of LVNC during labor is an ongoing area of research, and the literature is limited to only a few case reports; however, several uneventful vaginal deliveries and cesarean sections have been reported [5]. Additionally, only a few case reports of ventricular arrhythmias and ICD discharges in pregnant patients have been reported [6].

Such challenges include decisions such as balancing the use of neuraxial techniques to appropriately manage the sympathetic response secondary to the stress of labor while avoiding a drastic sympathectomy‐related decrease in systemic vascular resistance. Moreover, such decisions should also consider general anesthesia in cases where there is severe compromise of left ventricular function, which allows for easier management of afterload and cardiac output.

## 2 Case Description/Examination

A female in her 30s with a history of CPVT and LVNC presented for induction of labor due to decreased fetal movement and vaginal bleeding, raising concerns for placental abruption. She was initially evaluated during adolescence following the sudden cardiac death of an uncle at age 35 during physical activity. At that time, her electrocardiogram (EKG) and cardiac examination were normal, and no further testing was performed.

**FIGURE 1:**
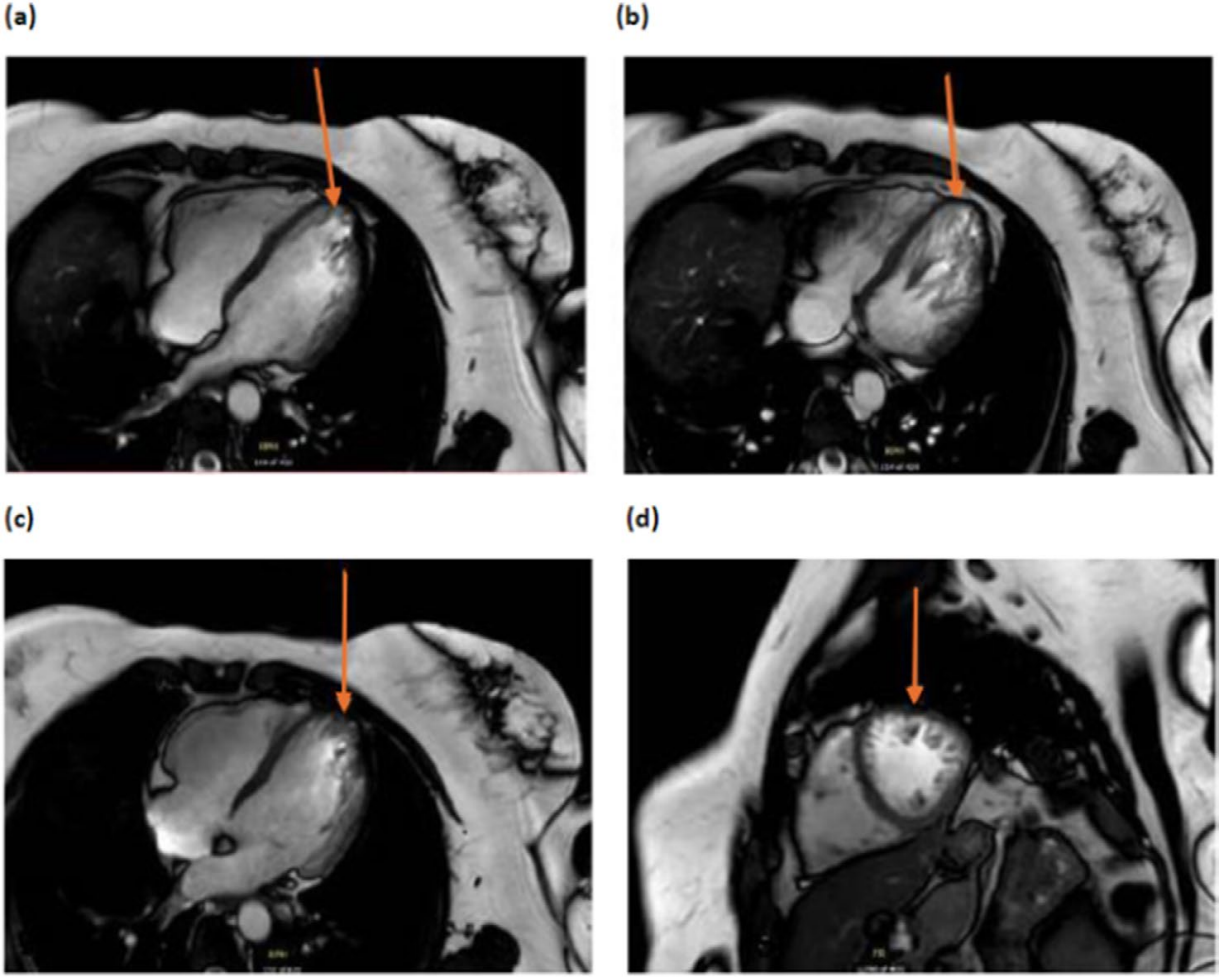
(a–c) Showing MRI coronal views of the left ventricle, (d) sagittal view of the left ventricle showing prominent apical trabeculations.

Subsequent genetic testing in her brother identified a pathogenic RYR2 deletion of exon 3, which prompted her evaluation by electrophysiology. She was diagnosed with CPVT and LVNC due to the genetic variant, a family history of sudden cardiac death, and exercise‐induced ventricular ectopy. Her clinical history included three syncopal episodes associated with palpitations, initially attributed to anxiety. Imaging MRI revealed increased left ventricular trabeculations (Figure 1) with preserved ejection fraction (62%) and mild aortic root dilation. She was managed with nadolol, titrated to 60 mg daily, which effectively controlled her ventricular ectopy during exercise.

Throughout her pregnancy, the patient was closely monitored by a multidisciplinary team. Serial echocardiograms showed stable ventricular function, and she was classified as NYHA Functional Class 1 Stage A heart failure. Recommendations for labor management included early epidural placement, careful titration of anesthesia, strict fluid management, and the continuation of beta‐blockade therapy.

## 3 Methods

### 3.1 Differential Diagnosis

The patient's presentation with vaginal bleeding and decreased fetal movement necessitated consideration of placental abruption. Additionally, CPVT and LVNC posed risks of hemodynamic instability, ventricular arrhythmias, and other complications triggered by the physical and emotional stress of labor.

### 3.2 Investigations

**FIGURE 2:**
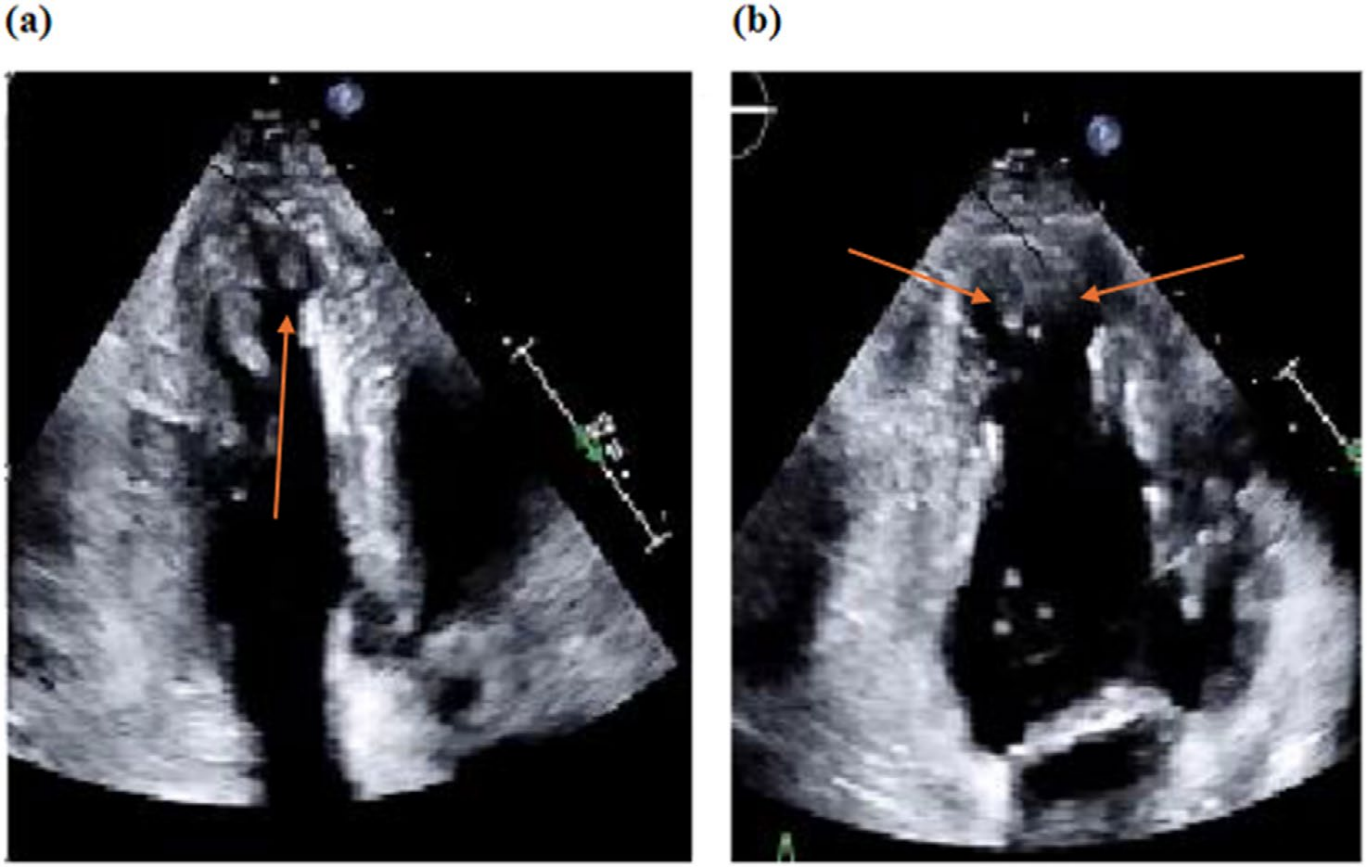
(a) 3 chamber (b) 2 chamber transthoracic echocardiogram showing apical trabeculations.

**FIGURE 3:**
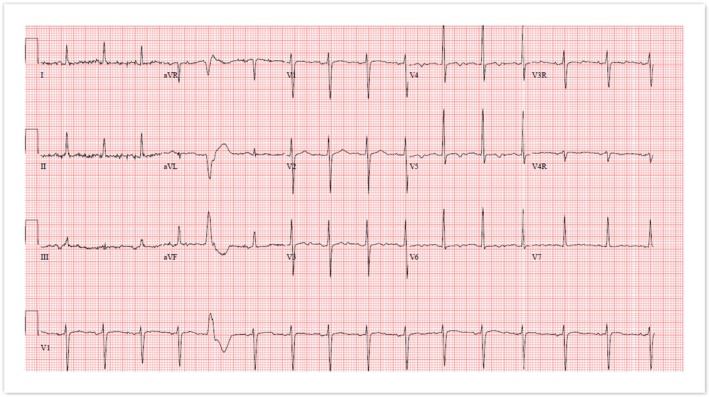
ECG showing normal sinus rhythm with one premature ventricular complex.

**FIGURE 4:**
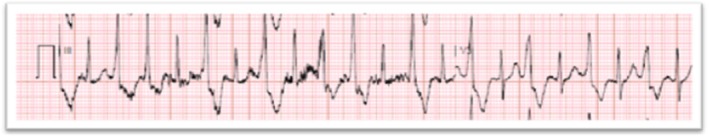
Rhythm strip of Exercise test showing Bigeminy.

The patient was monitored using continuous cardiac telemetry upon admission, with close observation of vital signs, including blood pressure, heart rate, and oxygen saturation. Echocardiography performed prior to delivery showed apical trabeculations (Figure 2) and stable ventricular function with an ejection fraction of 57%, with normal left ventricular diastolic function, normal right ventricular systolic function, with the left and right atrial cavities dilated, with the left ventricle normal in size. No significant valvular abnormalities were noted. Brain natriuretic peptide levels were within normal limits. EKG showed normal sinus rhythm with one premature ventricular complex (Figure 3). Exercise stress test showed no bi‐directional PVCs and only one four‐beat run of ventricular bigeminy near peak exercise with no ventricular ectopy during exercise (Figure 4).

### 3.3 Treatment

The anesthetic plan prioritized minimizing catecholamine surges. An epidural catheter was placed early during labor to reduce pain‐induced adrenergic stimulation. A slow infusion of bupivacaine 0.0625%‐fentanyl 2 mcg/mL was initiated at a rate of 10 mL/h. The use of an epinephrine‐containing test dose was avoided to prevent catecholaminergic effects.

When category 2 fetal heart tracing with prolonged deceleration necessitated an urgent cesarean section, the epidural was titrated to a surgical level using 20 mL of 2% lidocaine with 100 mcg fentanyl. Post‐delivery analgesia included 8 mL of 0.5% bupivacaine and 2.5 mg preservative‐free morphine sulfate. Vasopressor support with phenylephrine was utilized to manage hypotension, avoiding agents such as ephedrine. Strict fluid limitations (< 2 L per day) were set by the obstetric team. Multidisciplinary collaboration among obstetricians, anesthesiologists, and cardiologists ensured comprehensive care throughout the procedure.

## 4 Results (Outcomes and Follow‐Up)

The patient underwent an uncomplicated cesarean section and remained hemodynamically stable throughout the procedure, with a mean arterial pressure maintained between 80 and 100 mmHg and a heart rate in the mid‐80s. Delivery was uneventful, and postpartum vital signs, telemetry, and a repeat echocardiogram confirmed preserved ventricular function (ejection fraction 55%). Brain natriuretic peptide levels were within normal limits, and no arrhythmias or complications were noted during the immediate postpartum period.

The patient was discharged in stable condition with follow‐up instructions to continue care with cardiology and electrophysiology. At subsequent follow‐up visits, both maternal and neonatal outcomes remained favorable, and no cardiac events or complications were reported.

## 5 Conclusion

CPVT and LVNC are rare conditions that pose challenging management in the obstetric patient.

Careful anesthetic management of these patients requires specific goals to prevent catecholaminergic release and mitigate the risk of cardiac complications during the birthing process. These goals include but are not limited to the utilization of beta‐blockade, meticulous titration of epidural medication, avoidance of catecholaminergic inducing medications, and vigilant fluid management. Moreover, close coordination and communication between multidisciplinary teams are vital for optimal patient care.

## 6 Discussion

CPVT onset typically occurs in early childhood through teenage years but has occurred up to the fourth decade of life. The resting ECG is usually normal, including the QTc interval. The initial manifestation usually occurs with childhood syncopal episodes; however, it can also be silent, with the first presenting sign being sudden death in 10%–20% of patients. Correlation has been found between the age of first syncope and severity of the disease, with a worse prognosis in the case of early occurrence [2]. Diagnosis of CPVT is established in the setting of a normal resting EKG, with exercise‐or emotion‐induced ventricular tachycardia or with individuals with pathogenic gene variants. Numerous gene variants have been identified in CPVT patients. The RyR2 ryanodine receptor is the most noted gene alteration. However, other variants such as *CASQ2*, *TRDN*, *TECRL*, *KCNJ2*, *CALM1*, *CALM2*, and *CALM3* have been identified [1].

LVNC carries a high risk of complications including but not limited to malignant arrhythmias, thromboembolic events, and left ventricular dysfunction resulting in heart failure [7]. Both atrial and ventricular arrhythmias occur. Atrial fibrillation is the most common atrial arrhythmia, with supraventricular arrhythmias, AV nodal reentry tachycardia, and atrial flutter also occurring less frequently [8]. Ventricular tachyarrhythmias are much more common and occur in up to half of cases [9] including ventricular tachycardia and ventricular fibrillation.

There have been only a few case reports of ventricular arrhythmias in obstetric patients with CPVT which document ICD shocks in pregnancy. One of which required three shocks in the first trimester for polymorphic ventricular tachycardia. This patient was managed with metoprolol due to its safety profile and subsequently underwent a successful cesarean section under spinal anesthesia with no complications during or after delivery. The second case report involved a patient who had five ICD discharges at 26 weeks secondary to medication noncompliance. The patient was managed with propafenone and propranolol until delivery with no subsequent firing of her ICD during the remainder of her pregnancy and underwent an uncomplicated spontaneous vaginal delivery [6, 10]. In a previous study of cardiovascular causes of unexpected death during the peripartum period, SAD (sudden arrhythmic death) and underlying cardiomyopathies were the leading causes of death in 54% and 14% of patients, respectively [11] however, the risk of arrhythmic events during pregnancy in patients with CPVT remains largely unknown.

In one retrospective study, among 96 CPVT obstetric patients identified with a combined 228 pregnancies, there were only 6 cardiac events, mostly involving episodes of syncope and only one cardiac‐related death. Of note, among the 6 patients with events, none were taking beta blockers. The investigators did not find evidence that CPVT posed any additional risk of cardiac events in the pregnant or postpartum period versus nonpregnant patients. Moreover, following a diagnosis of CPVT, fetal and maternal outcomes were generally favorable, with the safety of pregnancy maintained in well‐controlled or minimally symptomatic obstetric patients with CPVT [12].

Main therapeutic options for treatment for CPVT include beta blockade (nadolol or atenolol is commonly used). Other case reports have suggested that Metoprolol may have increased side effects with greater patient discomfort experienced and may be inferior to atenolol, which offers a good safety profile. It is paramount for patients to adhere to a strict dosing regimen in every appointment with prenatal providers, as in their case report, missing one single dose resulted in symptomatic ventricular tachycardia requiring ICD therapy [6]. A lifesaving therapy for select patients is ICD placement, but caution is warranted as inappropriate shocks can lead to adrenergic stimulation and acute deterioration. Thus, ICD placement should be considered in refractory or recurrent cases [13]. Other treatment options for CPVT include anti‐arrhythmic medications (flecainide or propafenone). Invasive procedures such as left cardiac sympathetic denervation can also be considered [10].

Additionally, anesthetic considerations in an obstetric patient with CPVT should include avoidance of endogenous catecholamine surges secondary to anxiety or inadequate levels of anesthesia and analgesia, avoiding extrinsic catecholamines, and preparing to acutely manage ventricular tachycardia during the perioperative period [14]. A few case reports of pregnant patients with CPVT utilized cesarean section as the preferred delivery mode, but successful vaginal delivery has been reported as well, with epidural placement and delivery via cesarean section may be more useful in states of maternal or fetal compromise [10]. Pohjavuori's [15] study found no increased levels of norepinephrine and epinephrine in cord blood between infants born vaginally versus cesarean section. However, other studies have reported higher incidences of catecholamines in infants via cesarean section [16]. Regardless of mode of delivery, multidisciplinary collaboration and strict medication compliance are needed.

It is important to note that although CPVT is dangerous and requires the utmost care and careful planning, this condition is not a contraindication to future pregnancies. In fact, in a large cohort study, it was found that pregnancy is not associated with an increased risk of CPVT associated arrhythmias. However, the risk for exacerbation is heightened when antiarrhythmic therapy is decreased or stopped during pregnancy. Expert guidelines agree that multidisciplinary teams with extensive knowledge of CPVT should follow and counsel patients on proper antiarrhythmic drugs, possible ICD usage, as well as planning for the elevated adrenergic period of labor and delivery itself [17, 18].

Our case involved early placement of an epidural catheter to avoid catecholamine release secondary to labor contraction pain. If bupivacaine is selected as the epidural medication (as occurred in our case), slow titration of bupivacaine is warranted due to its potential for cardiotoxicity and increased risk of ventricular arrhythmias. Alternatively, ropivacaine should be considered as an alternative option for epidural medication selection. Additionally, during initial epidural placement, consideration should be made to possibly avoid a test dose with epinephrine due to its catecholaminergic effects. Moreover, strict fluid intake is necessary to avoid fluid overload and prevent heart failure exacerbation. To treat hypotension, pressor support should be given instead of fluid, and phenylephrine should be utilized instead of ephedrine due to its inherent mechanism of action as a sympathomimetic amine or epinephrine for similar reasons. Beta blocker medication should be continued throughout pregnancy. Lastly, multidisciplinary support is necessary to adequately manage these patients, including consultation with cardiology, electrophysiology, MFM, anesthesiology, etc.

## Author Contributions

**Chase Jackson:** conceptualization, data curation, investigation, methodology, project administration, resources, writing – original draft, writing – review and editing. **Jibran Ikram:** conceptualization, data curation, formal analysis, investigation, methodology, project administration, resources, software, validation, visualization, writing – original draft, writing – review and editing. **Jose L. Diz Ferre:** formal analysis, investigation, resources, visualization, writing – original draft. **Sabry Ayad:** conceptualization, data curation, formal analysis, investigation, methodology, project administration, software, supervision, validation, visualization, writing – original draft, writing – review and editing.

## Consent

Written informed consent was acquired from the patient whose clinical images and case details are written in the study to publish this report in accordance with the journal's patient consent policy.

## Data Availability

The data supporting this report's findings are available on request from the corresponding author. The data is not publicly available due to privacy or ethical restrictions.
